# Association of Tumor Budding With Immune Evasion Pathways in Primary Colorectal Cancer and Patient-Derived Xenografts

**DOI:** 10.3389/fmed.2020.00264

**Published:** 2020-07-03

**Authors:** Silvia Guil-Luna, Rafael Mena, Carmen Navarrete-Sirvent, Laura María López-Sánchez, Karima Khouadri, Marta Toledano-Fonseca, Ana Mantrana, Ipek Guler, Carlos Villar, Cesar Díaz, Francisco Javier Medina-Fernández, Juan Rafael De la Haba-Rodríguez, Enrique Aranda, Antonio Rodríguez-Ariza

**Affiliations:** ^1^Instituto Maimónides de Investigación Biomédica de Córdoba, Córdoba, Spain; ^2^Centro de Investigación Biomédica en Red de Cáncer (CIBERONC), Madrid, Spain; ^3^Unidad de Gestión Clínica de Anatomía Patológica, Hospital Universitario Reina Sofía, Córdoba, Spain; ^4^Unidad de Gestión Clínica de Cirugía General y del Aparato Digestivo, Hospital Universitario Reina Sofía, Córdoba, Spain; ^5^Unidad de Gestión Clínica de Oncología Médica, Hospital Universitario Reina Sofía, Córdoba, Spain; ^6^Departamento de Medicina, Facultad de Medicina de Córdoba, Universidad de Córdoba, Córdoba, Spain

**Keywords:** patient-derived xenografts, tumor budding, colorectal cancer, immune evasion, toll-like receptors, chemokines

## Abstract

Tumor budding has been found to be of prognostic significance for several cancers, including colorectal cancer (CRC). Additionally, the molecular classification of CRC has led to the identification of different immune microenvironments linked to distinct prognosis and therapeutic response. However, the association between tumor budding and the different molecular subtypes of CRC and distinct immune profiles have not been fully elucidated. This study focused, firstly, on the validation of derived xenograft models (PDXs) for the evaluation of tumor budding and their human counterparts and, secondly, on the association between tumor budding and the immune tumor microenvironment by the analysis of gene expression signatures of immune checkpoints, Toll-like receptors (TLRs), and chemokine families. Clinical CRC samples with different grades of tumor budding and their corresponding PDXs were included in this study. Tumor budding grade was reliably reproduced in early passages of PDXs, and high-grade tumor budding was intimately related with a poor-prognosis CMS4 mesenchymal subtype. In addition, an upregulation of negative regulatory immune checkpoints (PDL1, TIM-3, NOX2, and IDO1), TLRs (TLR1, TLR3, TLR4, and TLR6), and chemokine receptors and ligands (CXCR2, CXCR4, CXCL1, CXCL2, CXCL6, and CXCL9) was detected in high-grade tumor budding in both human samples and their corresponding xenografts. Our data support a close link between high-grade tumor budding in CRC and a distinctive immune-suppressive microenvironment promoting tumor invasion, which may have a determinant role in the poor prognosis of the CMS4 mesenchymal subtype. In addition, our study demonstrates that PDX models may constitute a robust preclinical platform for the development of novel therapies directed against tumor budding in CRC.

## Introduction

Tumor budding has recently received much attention in the setting of progression and invasion in several malignancies including colorectal cancer (CRC). Tumor budding is defined as a single tumor cell or cluster of up to 4 cells at the invasive front (1, 2). High-grade tumor budding is now established as an independent prognostic factor since it has been associated with shorter disease-free survival (DFS) and overall survival (OS) in several types of cancer (2–4). Currently, it is widely believed that tumor buds provide the histological basis for invasion and metastasis, but it is still a matter of controversy if it is directly related with the epithelial–mesenchymal transition (EMT) (5, 6).

High-grade tumor budding has been inversely correlated with the presence of immune infiltrate at the invasive front. In addition, an overexpression of stem-cell related genes as ZEB1, ZEB2, DES, and VIM, and the activation of both WNT and TGF-β signaling, has been demonstrated to be expressed in tumor buds (7–9).

In this context, tumor budding has been recently associated with the poor-prognosis CMS4 subtype of CRC (10). This mesenchymal-like subtype is characterized by overexpression of stem cell markers, neoangiogenesis, and activation of TGF-β and WNT/β-catenin pathways which modulate immune evasion and the metastasis process (11–13). CMS4 tumors display low content of immune cells and exhibited the worst DFS and OS, demonstrating an urgent need to develop therapies for this subtype (11, 12). These findings are in line with the described profiles of tumor buds. However, the potential relationship between the immunosuppressive microenvironment of this poor-prognosis subtype and tumor buds still remains unknown (7).

Recognizing that tumor budding is an important contributor of the tumor invasion prognosis and the close relation with the CMS4 subtype, the translation of tumor budding to preclinical models has major challenges. Patient-derived xenografts (PDXs) generated by direct engraftment of human tumor tissue into immunodeficient mice have emerged as powerful preclinical platforms for analysis of predictive biomarkers, therapeutic targets, and drug discovery in cancer (14).

In this study, firstly, we examined early passages of CRC PDXs as potential models to analyze tumor budding and, secondly, we elucidated a link between high-grade budding and CMS4 subtype and specific signatures of immune evasion. PDXs may greatly help in the understanding of tumor budding and the involved mechanisms in the tumor microenvironment, which will provide new strategies and prospects for more effective treatments. In addition, treatments which simultaneously tackle the interactions between tumor buds and surrounding stroma could more effectively kill tumor cells or at least limit tumor progression and metastatic dissemination.

## Materials and Methods

### Patient and Inclusion Criteria

A consecutive, population-based series of forty-five patients over 18 years of age with resectable colon cancer submitted to Reina Sofía Hospital (Córdoba, Spain) was prospectively included. To avoid the bias of neoadjuvant treated patients, all rectal cancer patients were excluded. The study was approved by the Reina Sofía Hospital ethical committee (Protocol number PI-0150-2017) in accordance with the Code of Ethics of the World Medical Association (Declaration of Helsinki). Informed consent was obtained from each patient, and clinical and pathological information was prospectively collected. The clinicopathological characteristics of patients are summarized in Table 1.

**Table 1:** Clinicopathological data of the patients included in the study.

	**All subjects, *n* (%)**
**Patients characteristics (*****n*** **=** **45)**	
Gender	
Female	15 (33%)
Male	30 (67%)
Age (mean ± SD)	73.8 ± 10.1
Distant metastasis at the diagnosis	
No	40 (89%)
Yes	5 (11%)
**Tumor characteristics (*****n*** **=** **45)**	
Tumor size (cm, mean ± SD)	4.2 ± 1.1
Tumor histological grade	
Low	39 (87%)
High	6 (13%)
TNM staging	
0	2 (4%)
I	1 (2%)
II	21 (47%)
III	16 (36%)
IV	5 (11%)
Anatomical location	
Left	20 (44%)
Right	25 (56%)
Histological subtype	
Well differentiated	6 (13%)
Moderately differentiated	34 (76%)
Poorly differentiated	5 (11%)
Mucinous component	
No	30 (67%)
Yes	15 (33%)
Stromal component	
<50%	14 (31%)
≥ 50%	31 (69%)
Inflammatory infiltrate	
Low	20 (44%)
Medium	16 (36%)
High	11 (25%)
Lymphatic invasion	
No	23 (51%)
Yes	22 (49%)
Perineural invasion	
No	26 (58%)
Yes	19 (42%)
Vascular invasion	
No	28 (62%)
Yes	17 (38%)
Molecular subtype	
CMS1	8 (18%)
CMS2/3	25 (58%)
CMS4	12 (24%)

### Processing of Tumor Samples and Establishment of PDX Models

A total of 45 tumor samples were obtained just after surgical resection. Three adjacent tumor pieces were immediately collected in sterile conditions. One tumor piece was snap frozen and stored in liquid nitrogen for gene expression profiling, another one was fixed in 4% buffered formalin and then embedded in paraffin (FFPE) for hematoxylin and eosin staining and IHC studies, and the third fresh tumor piece was included in sterile PBS and used for establishment of PDXs.

The PDX engraftment was performed according to Puig and coauthors in NOD-SCID mice (NOD.CB17-Prkdcscid/Rj) (Janvier Laboratory, Paris, France) of 4–6 weeks of age (15). The animals were fed with a standard diet (D03-SAFE, Augy, France) and provided with drinking water *ad libitum*. Mice were daily monitored, and tumor growth was weekly measured until tumor volume was 1 cm^3^. Mice were ethically sacrificed under isoflurane anesthesia followed by cervical dislocation when tumor reached that size, if they appeared to be suffering, or after 6 months without tumor growth. Samples were immediately collected, fresh-frozen, formalin-fixed, and reimplanted (P1) as described above. This process was repeated to produce subsequent passages (until P3). Animal care and experimental procedures were approved by the University of Córdoba Bioethics Committee and followed the regulations of the European Union normative (26/04/2016/066).

### Histological and Immunohistochemical Analysis of Patient Tumor Samples and PDX Models

Hematoxylin and eosin-stained tissue sections were evaluated by 2 trained pathologists (CVP and SGL) for the following criteria: histological subtype, invasion (lymphatic, vascular, or perineural), and stromal and inflammatory component. The degree of inflammatory cell infiltration was assessed in the center of the tumor and invasive margin of the tumor as reported previously (16). For the analysis of stroma, a representative 10 × magnification area of the invasive margin was selected and the percentage of the stroma for each sample was calculated as described by Gujam et al. (17).

On the other hand, IHC staining was performed on 4-μm FFPE sections using the antibodies detailed in Supplementary Table 1 for molecular classification of patient tumors and PDXs. Tissue sections were incubated in 10 mM citrate buffer (pH 6.0) for 5 min at 120°C for antigen retrieval. Endogenous peroxidase was neutralized by using the EnVision FLEX peroxidase-blocking reagent (Dako, Glostrup, Denmark) for 10 min. After blocking with 3% bovine serum albumin or following mouse-on-mouse staining protocol (Abcam, Cambridge, UK) in the case of PDXs, sections were incubated with the primary antibodies overnight at 4°C. Then, after incubation with the corresponding EnVision FLEX+ mouse or rabbit linker (Dako, Glostrup, Denmark) (30 min at room temperature), sections were incubated for 1 h with the secondary antibody EnVision FLEX/HRP (Dako, Glostrup, Denmark). The staining was visualized using 3,3-diaminobenzidine chromogen (Dako, Glostrup, Denmark) and counterstained with Harris hematoxylin. Negative controls without incubation with primary antibodies were also performed.

### Immunohistochemistry-Based Molecular CMS Classification

Molecular classification by IHC was performed as described elsewhere (18). Individual cores were scored by trained pathologists (CVP and SGL) for FRMD6, ZEB1, HTR2B, AE1AE3, and CDX2 intensity and content. For MSI status, an analysis was performed with specific antibodies against hMLH1, hMSH2, hMSH6, and hPMS2, as described above. Immunohistochemical scores for each antibody were entered in the online classification tool (crclassifier.shinyapps.io/appTesting/) as described elsewhere (18). Using this classification, tumors were classified as CMS1, CMS2/3, or CMS4 subtypes.

### Tumor Budding Determination

Tumor budding was defined as single tumor cells or tumor cell clusters of up to four cells in the stroma of the invasive front as previously reported (1). Tumor buds were assessed on pan-cytokeratin (clone AE1/AE3) immunostaining in a single hot spot measuring 0.785 mm^2^ for more accurate identification in cases of obscuring factors like inflammation or reactive stroma. Cutoffs as defined by International Tumor Budding Consensus Conference (ITBCC) were used: low (BD1, 0–4 buds; intermediate (BD2), 5–9 buds; and high (BD3) ≥10 buds (18).

### Immune Gene Expression Profiling of Patient Tumors and PDX Models

The expression of genes encoding molecules involved in immune checkpoints, Toll-like receptors (TLRs), and chemokine receptors and their ligands was analyzed using the nCounter PanCancer immune-profiling panel from NanoString (Seattle, WA, USA) both in patient tumors and their corresponding PDXs. In order to minimize the variability between patients and xenografts, P0 passage was used for immune gene expression profiling. For this purpose, total RNA extraction was performed using RNeasy Mini Kit (Qiagen, Hilden, Germany) following the manufacturer's recommendations. Quantification and determination of the RNA purity were performed using a NanoDrop™ 1000 spectrophotometer (NanoDrop® ND-1000 UV-Vis Spectrophotometer, NanoDrop Technology), and RNA integrity Number (RIN) was measured using an Agilent 2200 TapeStation equipment. Data analysis was performed using nSolver software (NanoString Technologies, Seattle, WA, USA) to manage the raw data generated from the expression of each gene (19). The positive or negative expression of one particular gene indicates that the number of RNA molecules is higher or lower than the mean, respectively.

### Statistical Analysis

Data were analyzed using GraphPad Prism 7 and R Software (version 3.5.0). Previously, in order to assess normality of the data, D'Agostino and Pearson Normality test was performed. The clinicopathological data were compared using Fisher's exact test or Mann–Whitney's test for qualitative and quantitative variables, respectively. Multivariate regression analysis was carried out with multinomial regression model for budding grades, including the variables selected by using the Akaike information criterion (AIC) with step-wise model selection. Differences in disease-free survival (DFS) were expressed as hazard ratios (HR) with 95% confidence intervals, and survival curves were constructed using the Kaplan–Meier method. All *p* values ≤ 0.05 were considered statistically significant.

## Results

### Tumor Budding Is Robustly Recapitulated in PDX Models and Is Closely Associated With the CMS4 Molecular Subtype of CRC

Overall, 82% (37/45) tumors were successfully engrafted with a mean latency period (time from day of inoculation to palpable tumor) of 30.7 ± 26.9 days for P0, which was shortened in subsequent passages (15.1 ± 9.8 for P1, 10.7 ± 5.0 for P2, and 8.1 ± 3.0 for P3).

**Figure 1:**
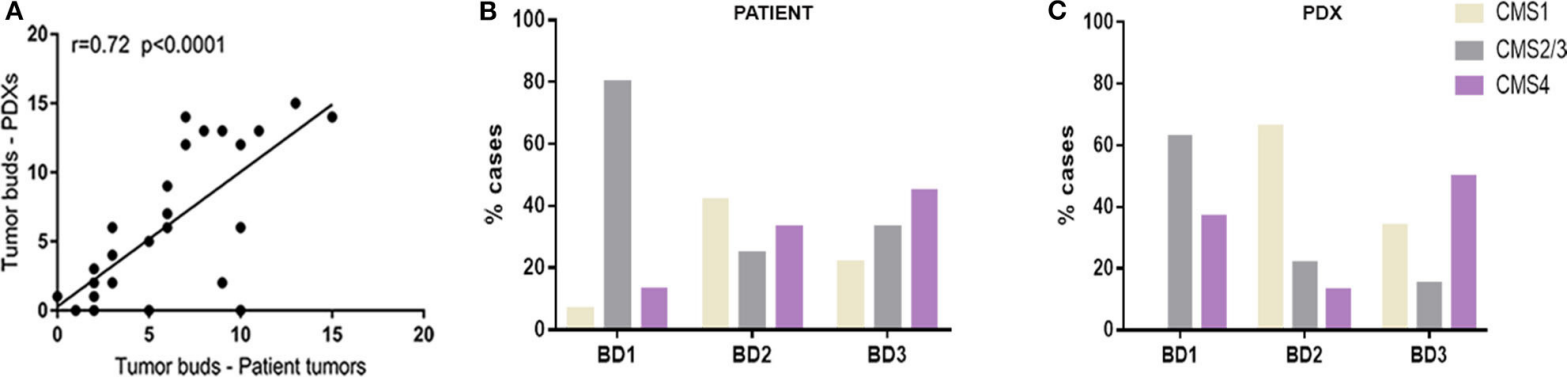
**(A)** Correlation between number of tumor buds in clinical tumors and in their corresponding PDX models. **(B)** Distribution of CMS molecular subtypes according to tumor budding grade in patient tumors. **(C)** Distribution of CMS molecular subtypes according to tumor budding grade in xenograft models (PDX).

Histopathological analysis of clinical tumors and their corresponding PDXs showed the preservation of the general tumor architecture and the histological subtype over several passages (Supplementary Figure 1). Remarkably, the determination of tumor budding status revealed a strong correlation between patient tumors and xenograft models (*r* = 0.72, *p* < 0.001) (Figure 1A).

**Figure 2:**
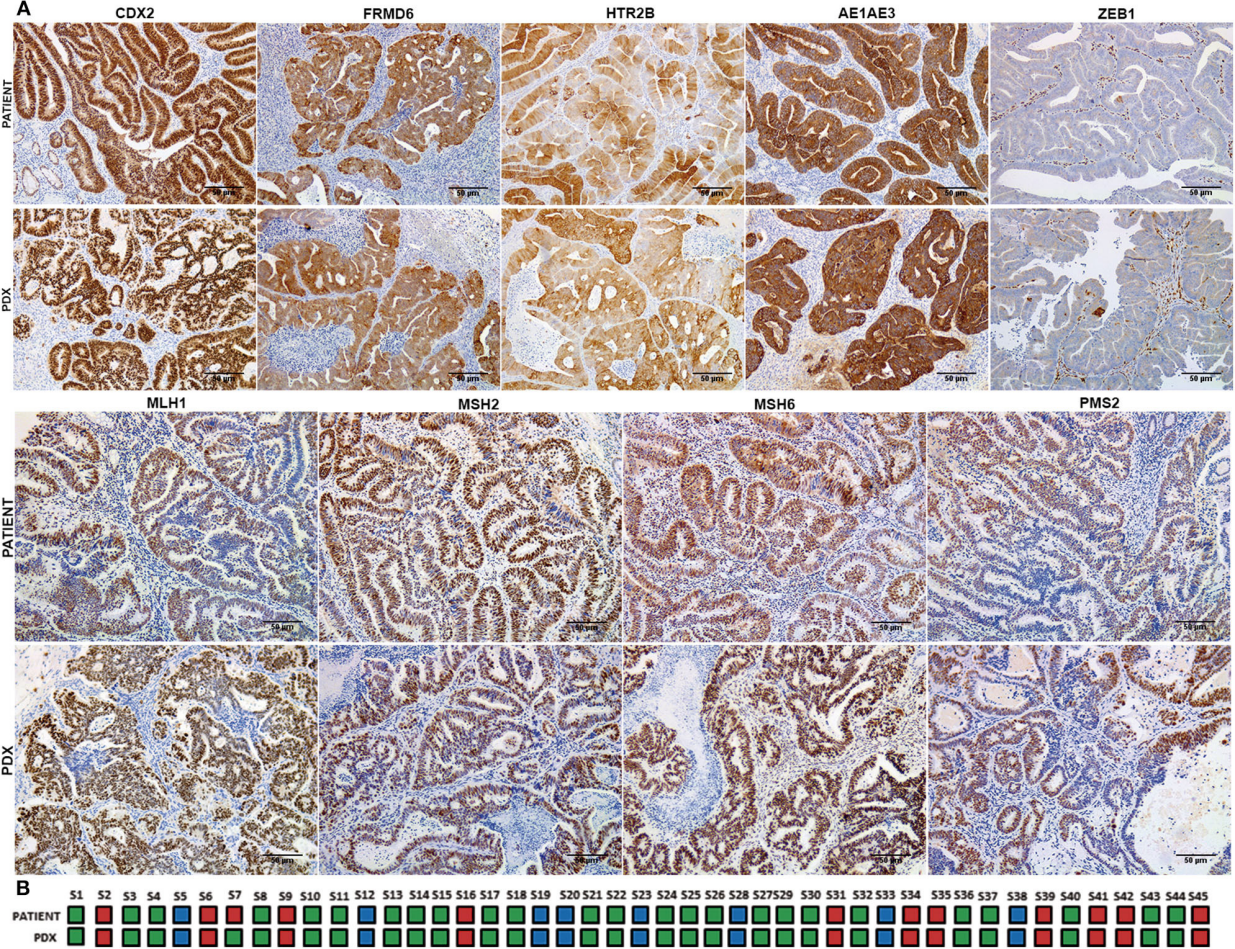
Immunohistochemical classification into CMS subtypes of patient tumor samples and their corresponding PDX models. **(A)** Representative immunohistochemical staining for CDX2, FRMD6, HTR2B, AE1AE3, ZEB1, MLH1, MSH2, MSH6, and PMS2 of a clinical tumor and its corresponding PDX model. **(B)** CMS classification concordance between patient tumors and their corresponding PDX models. Blue color corresponds to CMS1 subtype, green color corresponds to CMS2/3 subtype, and red color corresponds to CMS4 subtype. Scale bars: 100 μm.

In order to analyze the relationship between tumor budding and molecular subtypes of CRC, a molecular classification of patient tumors and xenografts was performed following the IHC-based method implemented by Trinh et al. (18). A strong concordance in the IHC expression patterns and consequently with the molecular CMS subtypes was observed between patient tumors and their corresponding PDXs with a Cohen's kappa coefficient of 0.96 (Figure 2A). Just in one case did the molecular subtype in the patient tumor (CMS4) shift to a different subtype (CMS2/3) in its PDX model (Figure 2B).

In particular, while most of the BD1 tumors (80% in tumor patients and 63% in PDXs) were classified as CMS2/3 subtype, BD3 tumors were more abundantly present in the poor-prognosis CMS4 subtype in both patient tumors and xenografts (Figures 1B,C). In addition, only 13% of human CMS4 subtypes were classified with low grade of tumor budding (BD1).

###  High-Grade Budding (BD3) Is Associated With Adverse Clinicopathological Factors

**Figure 3:**
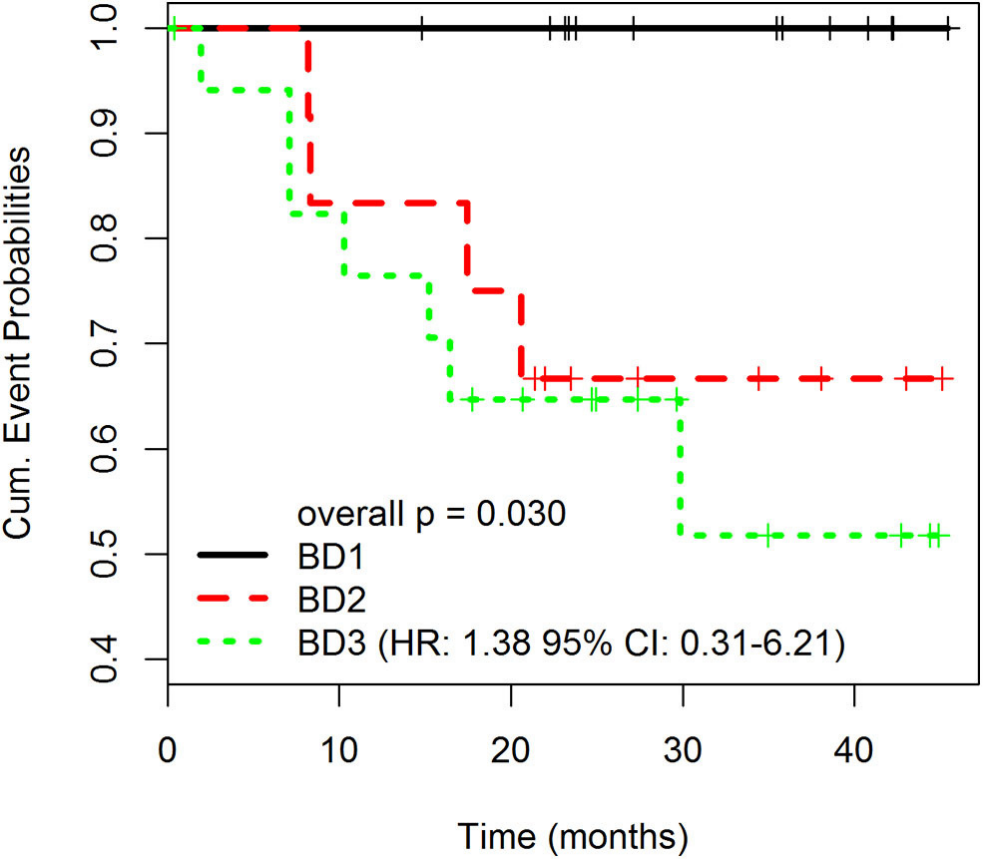
Disease-free survival (DFS) rates according to different grades of tumor budding (BD1, BD2, BD3). Overall *p* is 0.030. The p-values for pairwise comparisons are BD1 vs. BD2 = 0.034; BD1 vs. BD3 = 0.022; BD2 vs. BD3 = 0.595. HR: 1.38 95% CI: 0.31–6.21).

Table 1 summarizes clinicopathological characteristics of patients included in this study. A high-grade tumor budding (BD3) was identified in 18 (40%) patients, followed by 12 (27%) patients with BD2 tumor budding and 15 (33%) patients with low-grade budding (BD1). The relationship between tumor budding and clinicopathological characteristics of patients is presented in Table 2. On univariate analysis, high-grade tumor budding was associated with poorly differentiated carcinomas (*p* = 0.02), higher stromal component (*p* = 0.02), tumor vascular invasion (*p* = 0.005), and presence of distant metastasis (*p* = 0.02). The histological subtype, tumor size, and stromal component were entered as covariates into the final multivariate model, based on the variable selection with the Akaike information criterion (AIC) using stepwise selection (Table 3). Regarding survival analysis, no event data (disease progression) were observed in low-grade budding. The intermediate- and high-grade tumor budding (BD2 and BD3) was significantly associated with poor DFS (*p* = 0.03) when compared with low-grade budding (Figure 3). Additionally, survival probability of intermediate- and high-grade tumor budding was compared but no significant difference was found [HR: 95% CI De-long BD3 vs. BD2: 1.38 (0.31–6.21)] (Figure 3).

**Table 2:** Association between clinicopathological data of tumors and budding grade on univariate analysis.

**Parameters**	**BD1**	**BD2**	**BD3**	***P*-value**
**Patients' characteristic (*****n*** **=** **45)**				
Age (years, mean ± SD)	74.07 ± 10.33	75.50 ± 10.34	72.50 ± 10.19	0.7
**Tumor's characteristic (*****n*** **=** **45)**				
Tumor grade				
Low	14	11	14	0.35
High	1	1	4	
TNM staging				
0–I–II	11	6	7	0.13
III–IV	4	6	11	
Anatomical location				
Left	8	4	8	0.58
Right	7	8	10	
Histological subtype				
Well differentiated	5	0	1	0.02
Moderately differentiated	10	11	13	
Poorly differentiated	0	1	4	
Mucinous component				
No	10	10	10	0.28
Yes	5	2	8	
Inflammatory infiltrate				
Low	7	7	4	0.28
Medium	5	2	9	
High	3	3	5	
Lymphatic invasion				
No	11	7	7	0.13
Yes	4	5	11	
Perineural invasion				
No	10	5	10	0.43
Yes	5	7	8	
Vascular invasion				
Yes	1	5	11	0.005
No	14	7	7	
Distant metastasis				
No	14	10	12	0.05
Yes	0	3	6	
Tumor size (cm, mean ± SD)	4.50 ± 1.26	3.75 ± 0.91	4.25 ± 1.10	0.07
Stromal component (%, mean ± SD)	23.33 ± 19.88	26.67 ± 18.74	41.67 ± 19.47	0.02
**PDX model's approach**				
Engraftment rate (%, cases)	80 (12/15)	92 (11/12)	83 (15/18)	0.69
Latency period (days, mean ± SD)	32.08 ± 35.50	35.40 ± 25.52	28.20 ± 22.53	0.16

*BD1, budding grade 1; BD2, budding grade 2; BD3, budding grade 3*.

**Table 3:** Multivariate analysis of clinicopathological data of the tumors.

**Variables**	**BD2 vs. BD1**			**BD3 vs. BD1**		
	**Coef (SD)**	***P*-value**	**OR 95% CI**	**Coef (SD)**	***P*-value**	**OR 95% CI**
Tumor size	−0.91 (0.46)	0.046	0.40 [0.16-0.98]	−0.73 (0.44)	0.097	0.48 [0.20–1.14]
Moderate vs. well diff.	12.33 (0.92)	<0.001	2.27 × 10^5^ [3.73 × 10^4^-1.38 × 10^6^]	1.90 (1.43)	0.184	6.72 [0.4–112.30]
Poorly vs. well diff.	23.12 (0.80)	<0.001	1.10 × 10^10^ [2.28 × 10^9^-5.38 × 10^10^]	13.66 (0.80)	<0.001	8.57 × 10^5^ [2.28 × 10^9^-5.38 × 10^10^]
Stromal component	0.007 (0.02)	0.772	1.007 [0.95–1.05]	0.04 (0.02)	0.055	1.04 [0.99–1.08]

*Coef, coefficient; SD, standard deviation; OR, odds ratio; CI, confidence interval; BD1, budding grade 1; BD2, budding grade 2; BD3, budding grade 3*.

###  Gene Expression of Immune Checkpoint, TLRs, and Chemokine Profiles Reveals Similar Distinct Patterns According to Tumor Budding Grade in Patients and Xenografts

By using a PanCancer immune-profiling panel from the NanoString platform, we identified those immune-related genes overexpressed in high-grade tumor budding compared with low-grade budding. In addition, the immune gene expression profiles of patient tumors were compared with the gene expression profiles of their corresponding xenograft models (P0).

**Figure 4:**
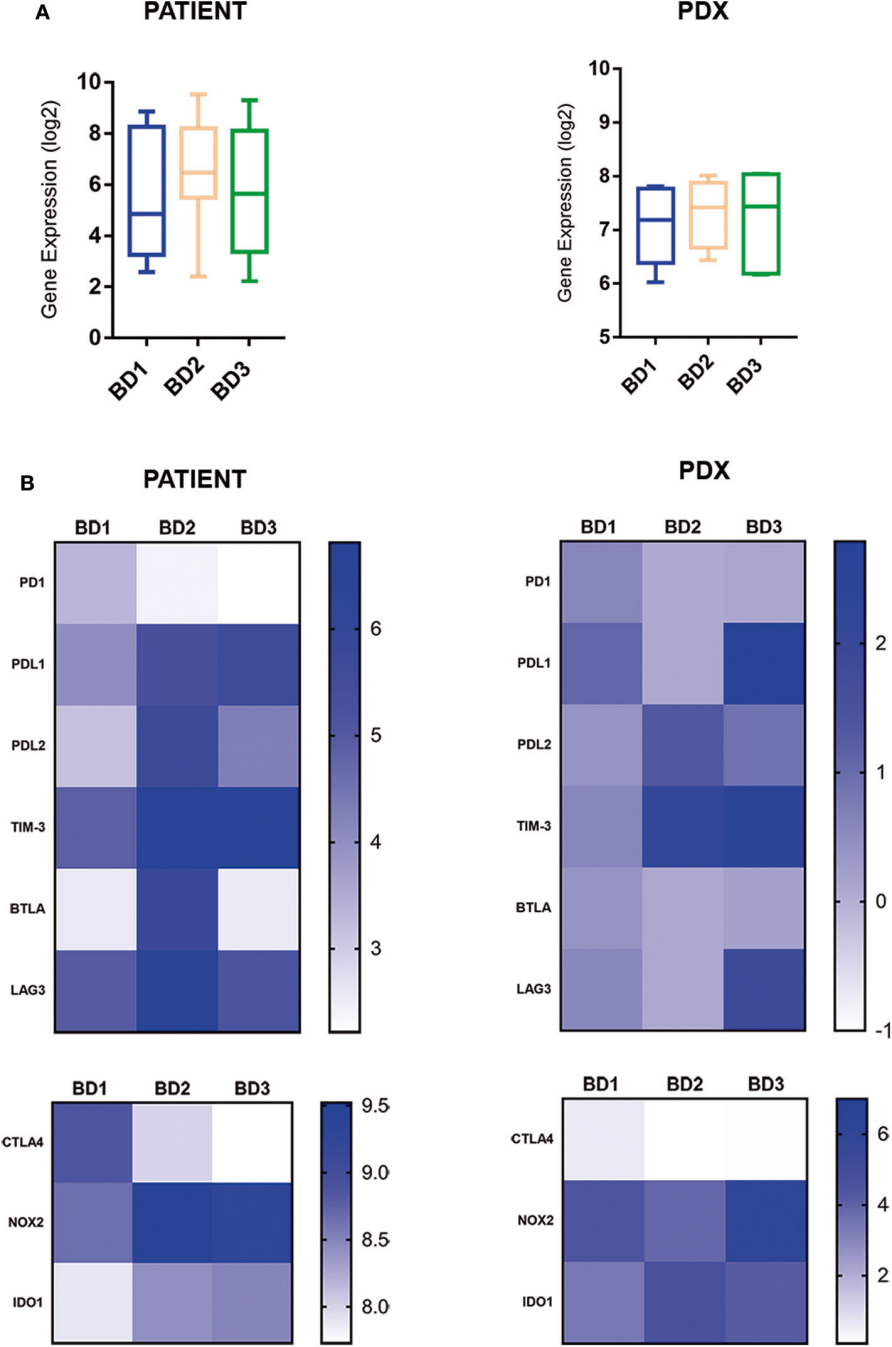
Global **(A)** and single **(B)** gene expression levels of immune inhibitor checkpoints in patient tumors and their corresponding PDX models, according to different grades of tumor budding (BD1, BD2, BD3).

The expression of inhibitory immune checkpoints according to tumor budding grade is displayed in Figure 4. The comparative analysis revealed a general upregulation of immune checkpoint-related genes in tumors with BD3 tumors in comparison with BD1 tumors (Figure 4). Interestingly, these immune signatures were remarkably preserved in their corresponding PDX models. Particularly, a higher expression of PDL1, TIM-3, NOX2, and IDO1 genes was observed in BD3 tumors. However, PD1 and CTLA4 genes were less expressed in the higher tumor budding grades in both patients and xenografts (Figure 4).

**Figure 5:**
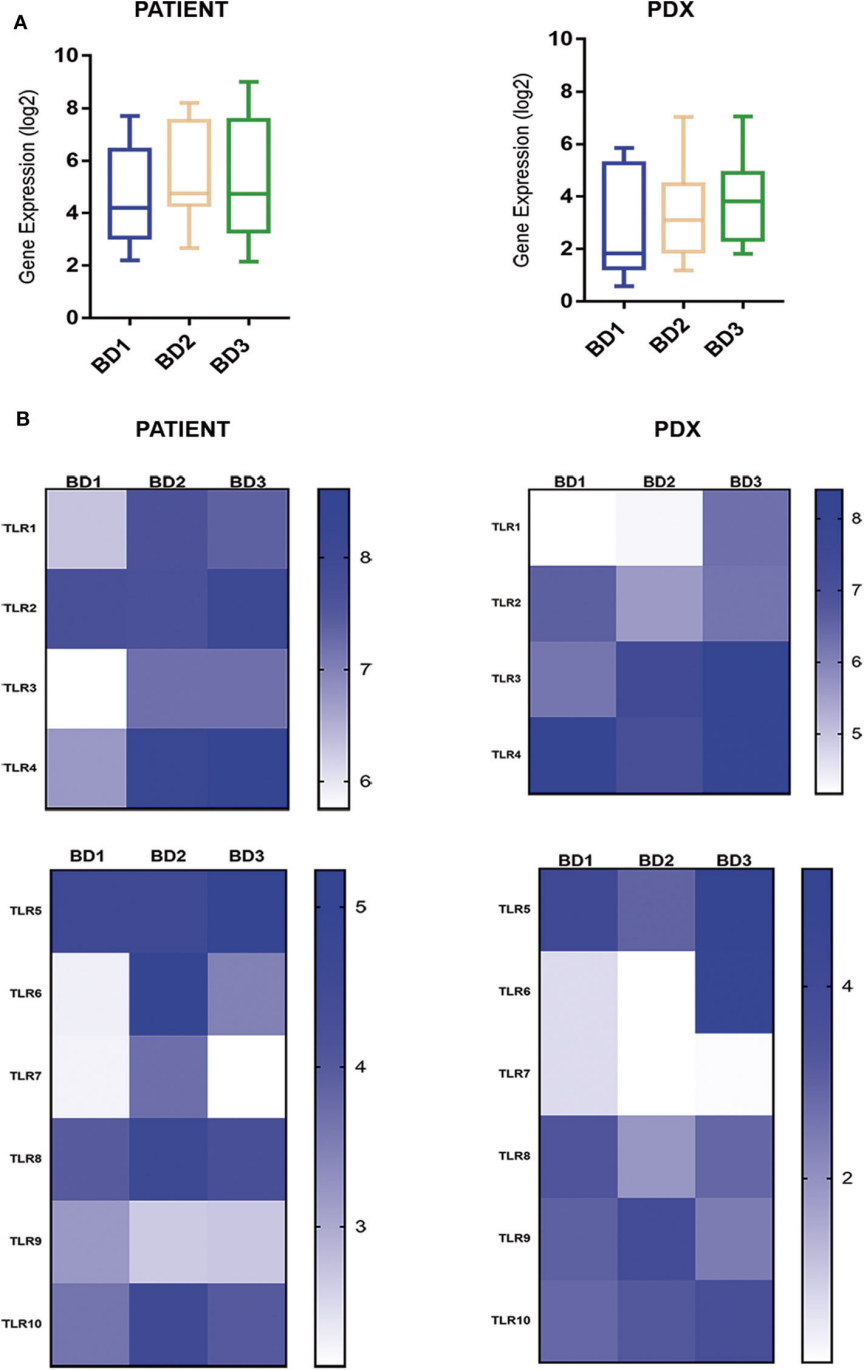
Global **(A)** and single **(B)** gene expression levels of the TLR gene family in patient tumors and their corresponding PDX models, according to different grades of tumor budding (BD1, BD2, BD3).

The expression of the TLR superfamily also displayed a high correspondence between patients and PDXs with the highest values for high-grade tumor budding (Figure 5). Of note, TLR1, TLR3, TLR4, and TLR6 were overexpressed in tumors with BD3 compared to BD1 tumors (Figure 5).

**Figure 6:**
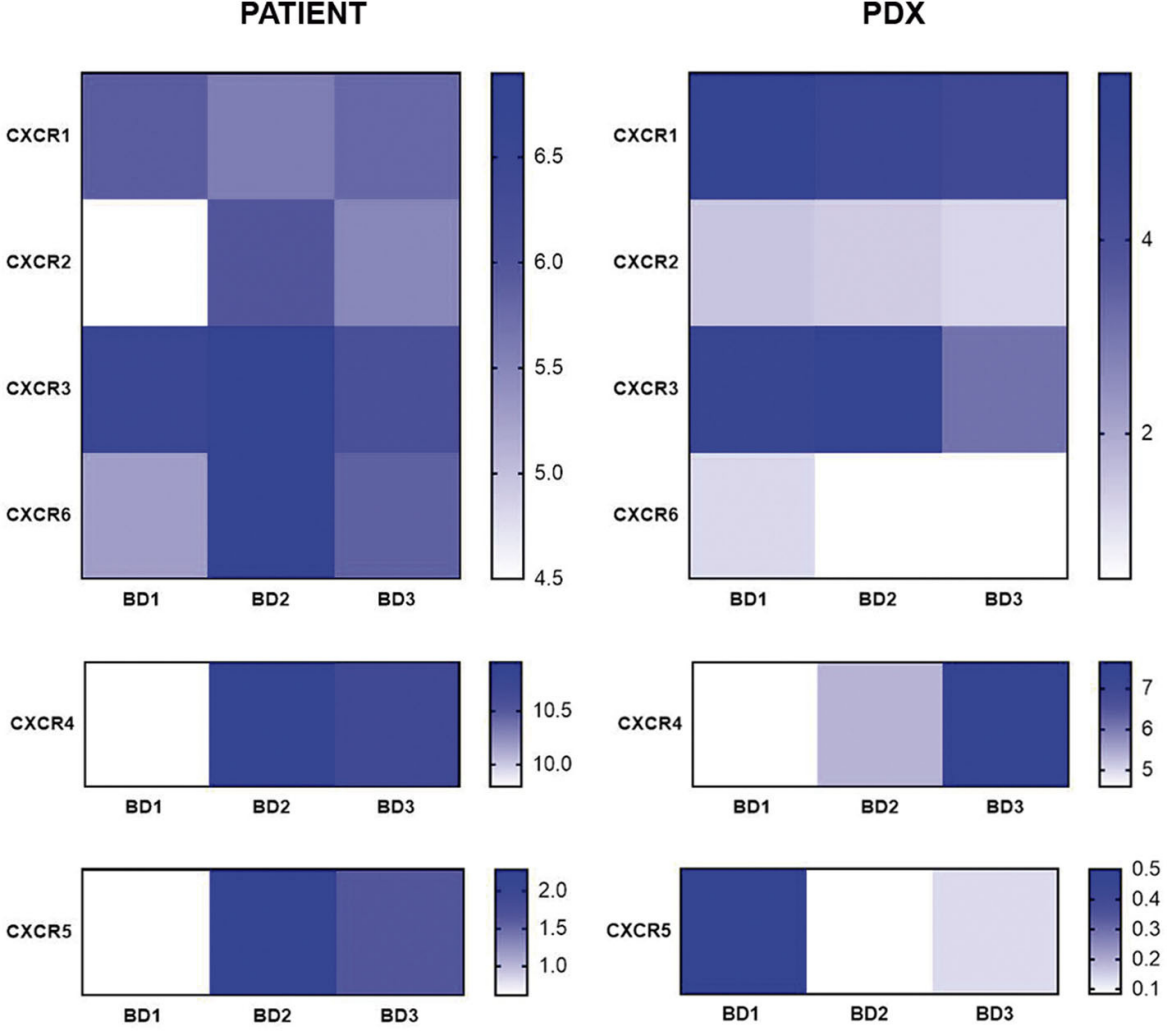
Gene expression of CX chemokine receptors in patient tumors and their corresponding PDX models, according to different grades of tumor budding (BD1, BD2, BD3).

**Figure 7:**
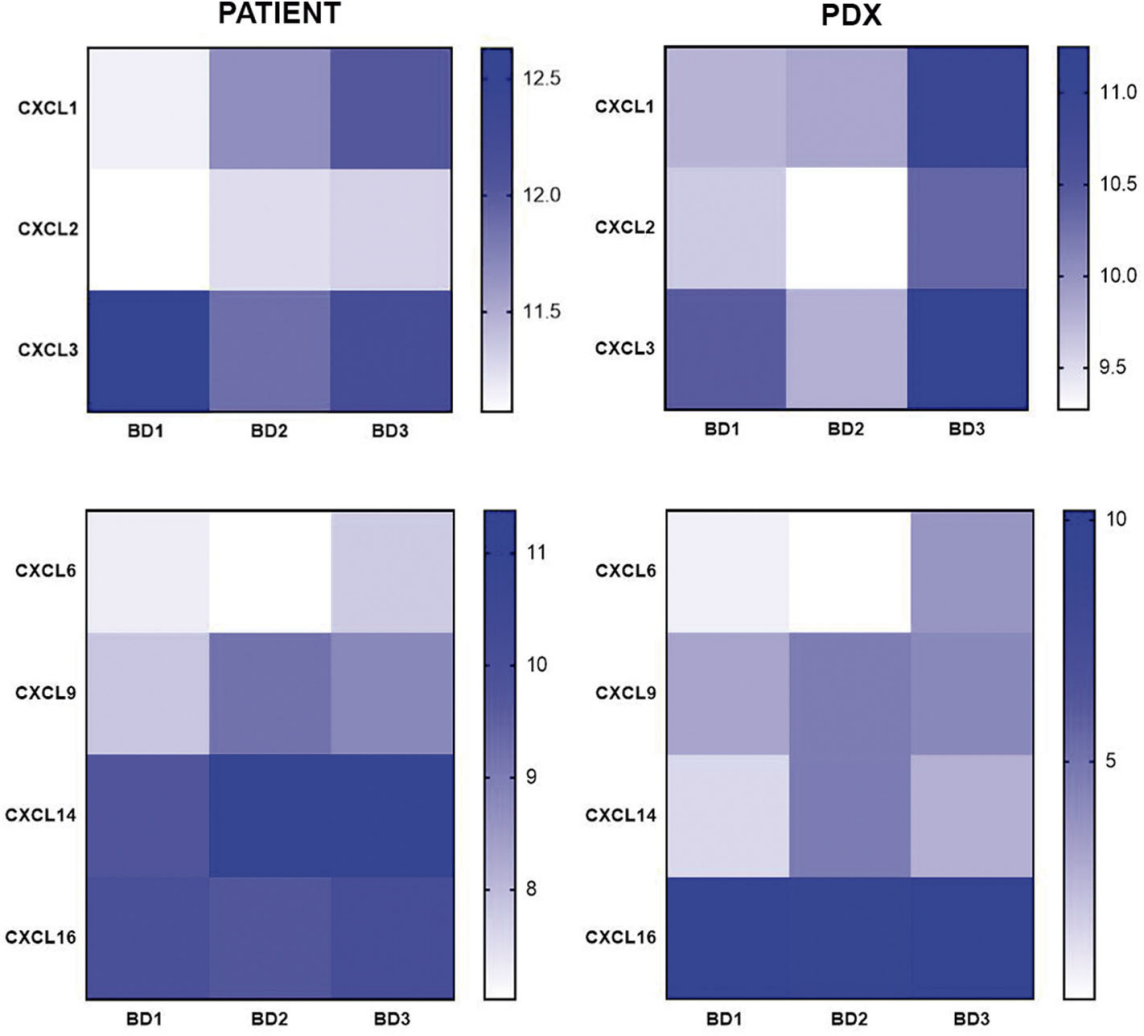
Gene expression of CX chemokine ligands in patient tumors and their corresponding PDX models, according to different grades of tumor budding (BD1, BD2, BD3).

Regarding the CX chemokine receptor family, the results showed that BD3 tumors were associated with a higher expression of CXCR2 and CXCR4 (Figure 6) than BD1 tumors were. Among the chemokine ligands, CXCL1, CXCL2, CXCL6, and CXCL9 genes also displayed a higher expression in tumors with BD3 compared to low-grade tumor budding (Figure 7). Notably, these distinct gene expression profiles of chemokine receptors and ligands depending on different budding statuses were in general, with some exceptions, preserved in the PDX models.

Despite the similar immune-related gene expression patterns found between primary tumors and PDXs with BD1 and BD3 grades, this correspondence was not as evident in PDXs with BD2 grade.

## Discussion

Taking into account that tumor heterogeneity is one of the major obstacles in the success of the new personalized therapies for CRC, preclinical platforms which faithfully represent the complex tumor biology are urgently needed. Furthermore, the significance of tumor budding as an independent prognostic factor has now been well established, reinforcing the notion that may constitute a promising target for cancer therapy. However, the interaction between tumor budding and the immune tumor microenvironment still remains unclear. The present study demonstrates that tumor budding is reliably reproduced in early passages of PDXs of CRC. Moreover, our data support that high-grade tumor budding is intimately connected with poor-prognosis CMS4 subtype and with specific gene signatures related to tumor immune evasion.

Our data confirmed that tumor budding is associated with adverse clinicopathological characteristics, such as tumor size, poor histological differentiation, vascular invasion, and poor outcome, as previously reported in several type of cancers (2, 7, 10, 20). An interesting finding of the present study was the high level of correspondence between the budding score in clinical tumors and their corresponding PDX models. Intriguingly, the immune deficiency in host mice did not promote an increase in tumor budding. Pu et al. (21) demonstrated that patient-derived immune cells coexist in the first and second passages with a survival rate of 290 days in the mouse. Recently, tumor budding has also been demonstrated in both center and the invasion front in CRC cell-line xenografts (22). These findings strongly suggest that early passages of PDXs preserve the distinctive cross talk between cancer cells and the immune microenvironment and determine the suitability of this preclinical platform as a model of tumor budding in CRC.

Recently, Trihn et al. (10) reported the potential association of high-grade budding with the CMS4 subtype of CRC in a series of CMS2/3 and CMS4 patient tumors. In the present study, we found similar results with the CMS4 subtype enriched by high-grade tumor budding compared with CMS1 and CMS2/3 subtypes. The fact that tumor buds are well-established independent adverse prognostic factors in CRC (1, 2) as well as the correspondence of CMS subtypes and tumor budding grade between PDXs and their human counterparts observed in our study supports the use of PDX models as powerful tools for the development of targeted therapies against mechanisms involved in tumor budding.

We found that high-grade budding was also significantly associated with stroma-rich tumors. Earlier reports in CRC and breast cancer have suggested an association between tumor budding and the presence of a high density of stromal myofibroblasts (23, 24). Tumor-associated stroma has been shown to facilitate EMT by inducing growth factors, which has been linked with higher capacity of migration and invasion of bud cells (24, 25). Thus, these findings highlight the potential role of the stroma in establishing a microenvironment supportive of the formation of tumor buds. Taken together, the budding phenotype seems to be associated with the high stromal component, which is also accentuated in the mesenchymal CMS4 subtype of CRC.

It is important to note the remarkable overexpression of inhibitory immune checkpoint-related genes (PDL1, TIM-3, NOX2, and IDO1) in BD3 tumors in comparison to BD1 observed in this study. All these upregulated genes have been previously related with tumor invasion and metastasis. However, limited studies have analyzed the expression of immune checkpoint genes in relation with tumor budding (26, 27). In agreement with our data, an upregulation of PDL1 expression has been reported in high-grade tumor budding of CRC suggesting that PDL1 might be specifically overexpressed during EMT to allow invasion and immune escape (27–29). On the other hand, TIM-3, which has been shown to inhibit antitumor immunity by mediating CD8 T-cell exhaustion and pathways involved in metastasis, is an emerging immune checkpoint in several cancers including CRC (30–32). IDO1 and NOX2 are known to exert a potent immunosuppressive effect in a variety of human solid tumors by reducing both tumor-infiltrating T cells as well as B cells (33, 34). Recent studies suggest that NOX2 knockdown reduces metastasis via mechanisms involving amelioration of immune-mediated clearance of metastatic tumor cells (33, 35). The overexpression of these inhibitory immune checkpoints in BD3 tumors observed in our study could explain the immune-permissive microenvironment that facilitates tumor bud formation, invasion, and progression even in early passages of PDXs. Nevertheless, PD1 and CTLA4 genes were more expressed in low tumor budding grade in both patients and xenografts. The distinct expression of PD1 and CTLA4 in immune cells and PDL1 in tumor cells, respectively, would explain these apparent contradictory findings. Hence, the high expression of PDL1 in BD3 tumors would be associated with the immune evasion mechanisms deployed by cancer cells at the invasive front in these tumors, while the overexpression of PD1 and CTLA4 genes in BD1 tumors would reflect their comparative higher immunogenicity. In this regard, the overexpression of these immune checkpoints has been observed in tumors with high immunogenicity and with good clinical response to anti-PD1 and anti-CTLA4 therapy (36, 37). Moreover, high PD1 expression has been recently reported to be associated with a favorable outcome in CRC patients while high-level PDL1 expression, either alone or in combination with PD1, was associated with a worse recurrence-free survival (38). The prognostic value of PD1 expression in lymphocytes and tumor cells and its interaction with PDL1 expression for the prognosis impact in CRC remain to be more deeply investigated. In this context, it may be plausible that CRC patients with low-grade budding will most likely benefit from anti-PD1 and anti-CTLA4 therapies.

TLRs are a diverse family of receptors that regulate gut inflammation but also found to be aberrantly expressed and associated with poor survival and with invasive and metastatic phenotypes in tumors (39, 40). In our study, TLR family expression, specifically TLR1, TLR3, TLR4, and TLR6, was upregulated in BD3 tumors in comparison with low-grade budding tumors (both in patient and PDX tumors) suggesting the presence of TLR-mediated alterations in the tumor invasive front. Overexpression of these TLRs has been previously detected in CRC (39–44). Although the specific mechanisms of TLR-mediated immune escape are still unknown, the current evidences indicated that the high expression of TLRs in tumors can contribute to tumor-cell resistance to apoptosis, malignant transformation of epithelial cells, and tumor progression (40). Results from our study support that TLR upregulation is closely related to BD3 of CRC, which marks them as promising targets for tumor therapy. In addition, it has been previously reported that the activation of TLRs is also accompanied by the expression of PDL1 in tumor cells and other inhibitory molecules as we have observed in this study (41).

Many cancer types show altered chemokine secretion profiles, favoring the recruitment of pro-tumorigenic immune cells such as myeloid-derived suppressor cells (MDSCs), tumor-associated neutrophils (TAN), tumor-associated macrophages (TAM), and regulatory T cells. Particularly, CXCR2 and CXCR4 are chemokine receptors for T-cells implicated in cancer invasion and metastasis (45, 46). Interestingly, these chemokines were overexpressed in BD3 tumors in patients and xenografts in our study. These two chemokine receptors play a crucial role in establishing the “pre-metastatic niche” for tumor cells and are now emerging as key players in the regulation of antitumor immunity (41, 47–50). In addition to these chemokine receptors, chemokine ligands such as CXCL1, CXCL2, CXCL5, CXCL6, CXCL8, and CXCL9 have been also significantly correlated with poor survival and metastasis in several cancers by recruiting MDSCs and suppressing the antitumoral activity of CD8+ T effectors cells. In agreement with these reports, our study reinforces the notion that many different chemokines contribute to antitumoral T cell recruitment and likely some of them may be related to the establishment of a pro-metastatic niche for the tumor buds.

Taken together, our data support a close association between TLRs, chemokines, and tumor budding, raising the exciting hypothesis that the activation of these immune targets may have a determinant role in tumor budding, especially in the case of the CMS4 subtype.

In summary, our findings support that tumor budding in CRC is strongly associated with the mesenchymal poor-prognosis subtype and the presence of a combination of immunosuppressive mechanisms to evade antitumor immunity. Besides, our study suggests that PDXs constitute robust preclinical platforms for reproducing CMS subtypes and tumor budding, hence allowing the development of novel challenging therapies directed against tumor budding in CRC, with special focus in the most aggressive CMS4 subtype.

## Data Availability Statement

The datasets have been uploaded to the repository, European Nucleotide Archive (ENA). The accession number is: PRJEB38274.

## Ethics Statement

The studies involving human participants were reviewed and approved by Reina Sofía Hospital ethical committee (Protocol number PI-0150-2017) in accordance with the Code of Ethics of the World Medical Association (Declaration of Helsinki). The patients/participants provided their written informed consent to participate in this study. The animal study was reviewed and approved by University of Córdoba Bioethics Committee and followed the regulations of the European Union normative (26/04/2016/066).

## Author Contributions

SG-L and RM performed most of the experiments, analyzed the data, and wrote the manuscript. CN-S, AM, KK, and LL-S helped to perform the patient-derived xenograft experiments. KK and MT-H helped to perform the CRC molecular classification. IG analyzed the statistical data of the manuscript. CV, FM-F, CD, and JD contributed to the collection and clinical characterization of human samples. AR-A and EA conceived the project, designed the experiments, and revised the manuscript. All authors contributed to the article and approved the submitted version.

## Conflict of Interest

The authors declare that the research was conducted in the absence of any commercial or financial relationships that could be construed as a potential conflict of interest.
