# Supplementary material for: Association of Tumor Budding With Immune Evasion Pathways in Primary Colorectal Cancer and Patient-Derived Xenografts
Source: Front Med (Lausanne). 2020 Jul 3;7:264. doi: 10.3389/fmed.2020.00264 (PMC7347987; doi:10.3389/fmed.2020.00264)
Supplement: Supplementary file 1 [file Table_1.docx]

Supplementary Material

## Supplementary Table

| **Primary Antibody** | **Host primary antibody** | **Dilution** | **Company** |
| --- | --- | --- | --- |
| FRMD6 | Rabbit polyclonal | 1:500 | Sigma Aldrich, MO, USA |
| ZEB1 | Rabbit polyclonal | 1:500 | Sigma Aldrich, MO, USA |
| HTR2B | Rabbit polyclonal | 1:75 | Sigma Aldrich, MO, USA |
| AE1AE3 | Mouse monoclonal | 1:500 | Thermo Scientific, IL, USA |
| CDX2 | Rabbit polyclonal | 1:500 | Novus Biologicals, Madrid, Spain |
| MLH1 | Mouse monoclonal | Ready to use | LEICA Biosystems, Warrington, UK |
| MSH2 | Mouse monoclonal | Ready to use | Master Diagnostica S.L., Madrid, Spain |
| MSH6 | Rabbit monoclonal | Ready to use | Master Diagnostica S.L., Madrid, Spain |
| PMS2 | Mouse monoclonal | Ready to use | Biocare Medical, CA, USA |

**Supplementary Table 1.** List of primary antibodies used for immunohistochemistry.
