# Supplementary figures and images for: Association of Tumor Budding With Immune Evasion Pathways in Primary Colorectal Cancer and Patient-Derived Xenografts
Source: Front Med (Lausanne). 2020 Jul 3;7:264. doi: 10.3389/fmed.2020.00264 (PMC7347987; doi:10.3389/fmed.2020.00264)

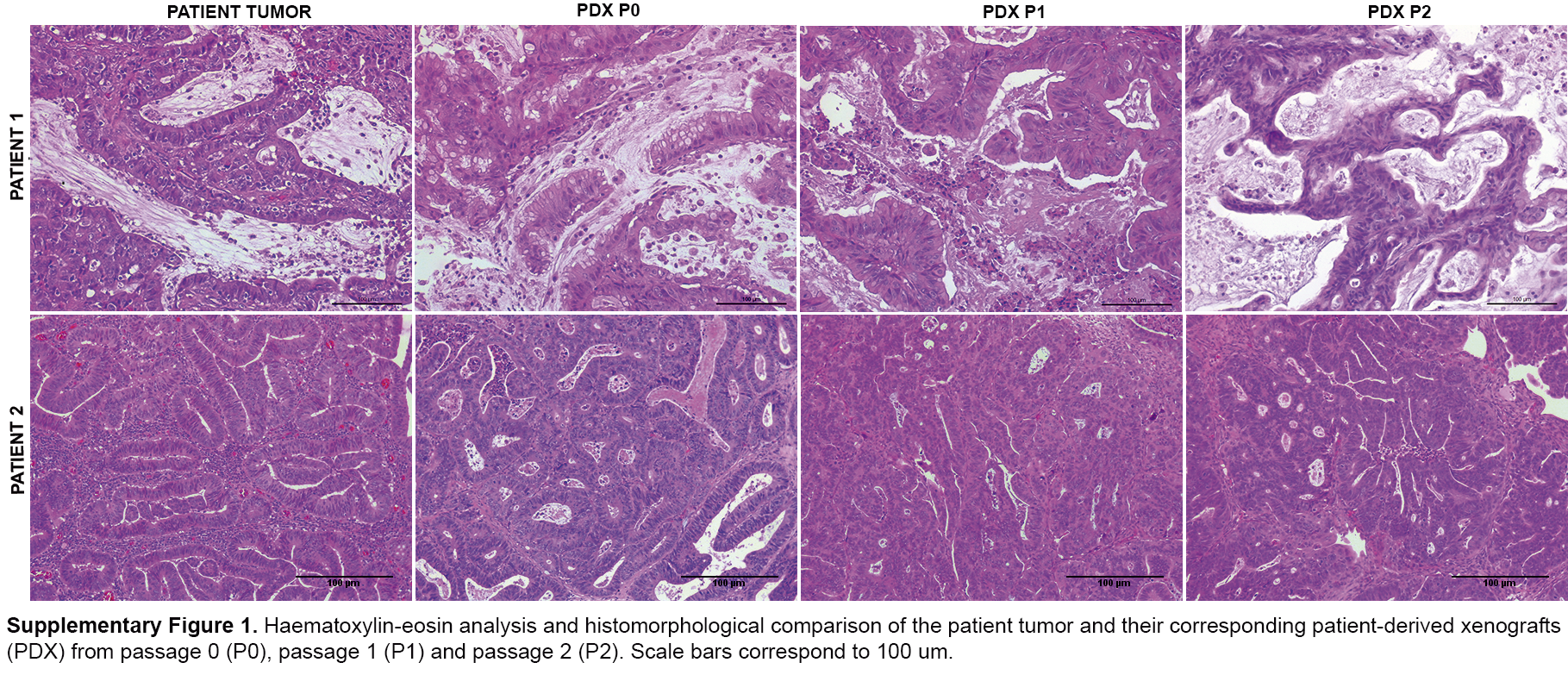

Supplement: Supplementary file 2 [file Image_1.tif]
